# Optimization and Application of Electrochemical Transducer for Detection of Specific Oligonucleotide Sequence for *Mycobacterium tuberculosis*

**DOI:** 10.3390/bios8030084

**Published:** 2018-09-11

**Authors:** Ricardo A. M. S. Corrêa, Filipe S. da Cruz, Cátia C. Santos, Thiago C. Pimenta, Diego L. Franco, Lucas F. Ferreira

**Affiliations:** 1 Laboratório de Eletroquímica e Nanotecnologia Aplicada, Instituto de Ciência e Tecnologia, Universidade Federal dos Vales do Jequitinhonha e Mucuri, Rodovia MGT 367, Km 583, 5000, Alto da Jacuba, Diamantina MG 39100-000, Brazil; ricardoamscorrea@gmail.com (R.A.M.S.C.); filipenq@gmail.com (F.S.d.C.); catiadtna@hotmail.com (C.C.S.); thiago.pimenta@ict.ufvjm.edu.br (T.C.P.); 2 Grupo de Eletroquímica Aplicada a Polímeros e Sensores, Laboratório de Eletroanalítica Aplicado à Biotecnologia e Engenharia de Alimentos, Instituto de Química, Universidade Federal de Uberlândia, Campus Patos de Minas, Av. Getúlio Vargas, 230, Patos de Minas MG 38700-128, Brazil; diegoleoni@ufu.br

**Keywords:** Electropolymerization, 4-hydroxyphenylacetic acid, oligonucleotides, electrochemical platforms, *Mycobacterium tuberculosis*

## Abstract

In this study, the electropolymerization of 4-hydroxyphenylacetic acid (4-HPA) over graphite electrodes (GE) was optimized, aiming its application as a functionalized electrochemical platform for oligonucleotides immobilization. It was investigated for the number of potential cycles and the scan rate influence on the monomer electropolymerization by using cyclic voltammetry technique. It was observed that the polymeric film showed a redox response in the region of +0.53/+0.38 V and the increase in the number of cycles produces more electroactive platforms because of the better electrode coverage. On the other hand, the decrease of scan rate produces more electroactive platforms because of the occurrence of more organized coupling. Scanning electron microscopy (SEM) images showed that the number of potential cycles influences the coverage and morphology of the electrodeposited polymeric film. However, the images also showed that at different scan rates a more organized material was produced. The influence of these optimized polymerization parameters was evaluated both in the immobilization of specific oligonucleotides and in the detection of hybridization with complementary target. Poly(4-HPA)/GE platform has shown efficient and sensitive for oligonucleotides immobilization, as well as for a hybridization event with the complementary oligonucleotide in all investigated cases. The electrode was modified with 100 cycles at 75 mV/s presented the best responses in function of the amplitude at the monitored peak current values for the Methylene Blue and Ethidium Bromide intercalators. The construction of the genosensor to detect a specific oligonucleotide sequence for the *Mycobacterium tuberculosis* bacillus confirmed the results regarding the poly(4-HPA)/GE platform efficiency since it showed excellent sensitivity. The limit of detection and the limit of quantification was found to be 0.56 (±0.05) μM and 8.6 (±0.7) μM, respectively operating with very low solution volumes (15 µL of probe and 10 µL target). The biosensor development was possible with optimization of the probe adsorption parameters and target hybridization, which led to an improvement in the decrease of the Methylene Blue (MB) reduction signal from 14% to 34%. In addition, interference studies showed that the genosensor has satisfactory selectivity since the hybridization with a non-specific probe resulted in a signal decrease (46% lower) when compared to the specific target.

## 1. Introduction

Organic compounds have been widely used for the development of organic thin films since the discovery of the polyacetylene polymerization by Shirakawa et al. in 1977 [1]. Generally, these thin films use conjugated monomers [2,3,4], since the resulting conjugated polymers contain highly delocalized *π* orbitals in their structure, which act as a medium for charge transport [5]. The continuity of these delocalized orbitals insures different electronic properties from those attributed to common polymers, such as low ionization energy, electrical conductivity, and high electronic affinity [6], and have been widely used as electrochemical platforms for biosensors development due to their charge transport properties and biocompatibility in a single structure [7].

These polymers have shown an excellent platform for biomolecules immobilization since they are relatively inexpensive materials, the production techniques are simple, they can be deposited onto different electrode materials and the choice of different molecular structures provide the construction of films with completely different characteristics [8,9]. Additionally, they provide a high surface area, adjustable morphology and a wide stability for the immobilized biomolecules [10]. This is justified since the polymers are presented as a three-dimensional matrix for the target, besides presenting characteristics of greater similarity to the environment properties of these biomolecules [11]. Generally, these polymers have preserved functional groups that are responsible for optimizing the interaction with the biomolecules (functionalized platforms).

Electrode surface modification with organic functional groups becomes increasingly relevant and has been used by many researchers to develop different electrochemical biosensors [10,12,13,14,15]. Through chemical control and surface coating, one can ensure reactivity, accessibility, and stability for the immobilized biomolecules, which are necessary conditions to achieve the biosensor major advantages: Sensitivity, selectivity, and stability. In this scenario, the surface modification with polymeric films has been used in the biosensors development to protect the electrode surface against the adsorption of interferents, to incorporate mediators and to provide biocompatibility [16]. The addition of groups such as amines, carboxylic acids, and thiols in these polymers has been reported to provide functionality for biomolecules immobilization onto modified surfaces [15,17,18,19,20,21]. Among the compounds already reported in the literature, the use of phenolic monomers is widely investigated, since the hydroxyl group attached to the aromatic ring can be oxidized to generate a radical cation, a fundamental species in the electrochemical polymerization process [22].

4-hydroxyphenylacetic acid (4-HPA) is a metabolic product of tyrosine [23] associated with the excessive growth of salivary and gastrointestinal bacteria, celiac disease [23,24,25], and cystic fibrosis [26]. Structurally, 4-HPA fits the desirable description mentioned before. It is a bifunctional monomer containing a hydroxyl (phenolic monomer) and a carboxyl group (functionality), and it has already been shown promising for the development of electrochemical platforms for biomolecules immobilization and biosensors development [22,27,28]. The mechanism is believed to occur via oxygen atoms leaving the carboxyl groups unchanged. The presence of this particular functional group can help the immobilization of biomolecules over the transducer and as a thin film; its polymer can help to prevent the non-specific adsorption of undesirable interferents. 

Usually, the experiments are performed after the electropolymerization to confirm the presence of the polymer and to characterization (electrochemical, spectroscopic, morphologic, etc.). However, just the presence of the polymer does not guarantee the applicability to sensor development. Thicker polymers may increase the diffusion layer to the bulk solution, hampering the electron transference, and thinner films may leave part of the electrode uncovered, which greatly decreases reproducibility and allows non-specific adsorption. Simple optimization parameters as the number of cycles and scan rate can influence directly in the polymer formation and its use for biomolecule immobilization. The different conformation and thickness of the material may present different scenarios to further interactions and are easily feasible. 

Tuberculosis (TB) is one of the oldest air-borne disease existing [29], with typical lesions containing acid-fast bacilli identified even in Egyptian mummies [30,31]. It is an infectious disease caused by the *Mycobacterium tuberculosis* bacillus, most responsible for the chronic infection and affects mainly the lungs. According to the World Health Organization (WHO) [32], TB is a worldwide problem affecting mainly the underdeveloped countries, predominantly in Asia and Africa, which concentrates more the 90% of the cases. 

The TB diagnosis is based on some classic methods as sputum culture, chest x-ray [33] and through nucleic acid probes, amplification tests, HPLC, gas/liquid chromatography, and automated systems for radiometric and non-radiometric detection of the growth of mycobacteria in liquid culture [34]. Despite the reliability, the tests demand time, some of the equipment is expensive and there is the need for skilled labor. Synthetic oligonucleotides based on studies performed on the bacteria can be used for genosensors development and are readily available. The easiness in the sensor assembly, the fast and real-time response, low-cost material, and the possibility to detect the disease at the initial stages of its development are pivotal features desired for health care improvement. 

Thus, in this work, poly-(4-HPA) parameters of electropolymerization (number of cycles and scan rate) were evaluated and optimized for the application of this electrochemical platform in the development of an electrochemical genosensor for the detection of a specific sequence of *Mycobacterium tuberculosis* genome.

## 2. Materials and Methods

### 2.1. Instrumentation

All electrochemical measurements were carried out on Potentiostat/Galvanostat from AUTOLAB^®^ model PGSTAT128N with a FRA32M model (EchoChemie, www.methrom.com.br) coupled to a microcomputer with NOVA 1.10 software.

Electrochemical experiments for polymeric film generation and characterization were performed in an electrochemical cell with 25.0 mL capacity, while all the biomolecules detection experiments were performed in an electrochemical cell with 1.0 mL capacity. Ag/AgCl (KCl 3.00 M) electrode was used as reference electrode. Graphite disk with 29.7 mm^2^ geometric area and platinum wire (1.0 cm^2^) were used as working and auxiliary electrode, respectively.

### 2.2. Materials

All chemical reagent used were of analytical grade. 4-HPA monomer (99%, Alfa Aesar, Haverhill, MA, USA); HClO_4_ (70%, Sigma-Aldrich, São Paulo, São Paulo, Brazil); K_3_Fe(CN)_6_ e K_4_Fe(CN)_6_ (99%, Sigma-Aldrich, São Paulo, São Paulo, Brazil); KCl (99%, Vetec, Duque de Caxias, Rio de Janeiro, Brazil); K_2_HPO_4_ e KH_2_PO_4_ (99%, Labsynth, Diadema, São Paulo, Brazil); Methylene Blue (98–103%, Vetec, Duque de Caxias, Rio de Janeiro, Brazil); Ethidium Bromide (97%, Vetec, Duque de Caxias, Rio de Janeiro, Brazil); and NaCl (99%, Vetec, Duque de Caxias, Rio de Janeiro, Brazil). Graphite bar (99.999%, Alfa Aesar, Haverhill, MA, USA).

poliGA-1 (5′-GGG GGG GGA AAA AAA A-3′), poliGA-2 (5′-GGA GAG GGG GAA AGA A-3′) oligonucleotides and their respective complementary targets poliCT-1 (3′-CCC CCC CCT TTT TTT T-5′) and poliCT-2 (3′-CCT CTC CCC CTT TCT T-5′) were obtained as lyophilized powder from Invitrogen^®^ and used as a primary probe for transducer optimization.

Probe (5′-CTC GTC CAG CGC CGC TTC GG-3′), target (5′-CC GAA GCG GCG CTG GAC GAG-3′) and interferent (5′-CA GAG GTG GCG ATG CAC CAT-3′) were used on the development of the biosensor to detect *Mycobacterium tuberculosis-*specific genome. The interferent presents 65% similarity to the target, and it was also obtained as a lyophilized powder from Invitrogen^®^.

All solutions were prepared prior to use using deionized water obtained from the PURELAB Classic DI (ELGA) water ultra purified. All solutions were deoxygenated, when necessary, with N_2(g)_ ultrapure for 10 min.

### 2.3. Electropolymerization and Optimization of the Functionalized Platforms

Prior to the electropolymerization process, the graphite electrodes (GE) were electrochemically conditioned according to the procedure described by Ferreira et al. [35].

The electropolymerization was conducted by cyclic voltammetry (CV), cycling the potential from 0.0 to +1.20 V. The number of potential cycles and scan rate has a direct influence on the film formation and they were investigated as followed: 25, 50 100, 150, and 200 cycles at 50 mV/s, the same scan rate as other electropolymerization studies [22,28,36]; and 25, 50, 75, and 100 mV/s with 100 cycles of potential.

4-HPA solution at 2.50 mM was prepared in 0.50 M HClO_4_ aqueous solution, which was used as a supporting electrolyte. After electropolymerization, the electrodes coated by poly(4-HPA), poly(4-HPA)/GE, which were washed with deionized water and dried under N_2(g)_ flow. 

The transductor was analyzed in a solution containing only the supporting electrolyte to verify the polymer adsorption, as well as its electrochemical activity. Therefore, five cycles of potential were carried out at the same electropolymerization range, in order to remove monomer that may have been “trapped” during the procedure.

### 2.4. Electrochemical Behavior and Scanning Electron Microscopy (SEM) of Poly(4-HPA)

Analyzes of GE and poly(4-HPA)/GE were conducted using potassium ferrocyanide/ferricyanide redox pair to study the charge transfer resistance at the electrodes surface. Thus, measurements by CV were conducted in 5.00 mM K_4_[Fe(CN)_6_]/K_3_[Fe(CN)_6_] aqueous solution containing 0.10 M KCl and the potential range was changed from −0.25 to +0.80 V.

The morphology of GE and poly(4-HPA)/GE were verified from the SEM images obtained using a model TM-3000 tabletop microscope from Hitachi. Images were obtained at 15 kV using the backscattered electrons detected using the composed mode.

### 2.5. Electrochemical Impedance Spectroscopy (EIS)

EIS analyzes were carried out in 5.0 mM K_4_[Fe(CN)_6_]/K_3_[Fe(CN)_6_] solution containing 0.10 M KCl. The frequency range was varied between 10^6^ to 10^−2^ Hz and the open circuit potential (OCP) was applied. In order to guarantee the linearity of the impedimetric response, the RMS amplitude of the sinusoidal perturbation was 10 mV (p/p). Kramers-Krönig transforms were applied and the system linearity was observed in all cases (χ^2^ < 10^−3^).

An equivalent circuit that best fits to the system proposed was used to analyze the experimental data. Simulation procedures were performed using software Nova 1.10 from Autolab.

### 2.6. Poly(4-HPA) as an Electrochemical Platform for the Oligonucleotides Immobilization

The immobilization of polyGA-1 and polyGA-2 probes were carried out by physical adsorption by dipping 20 μL of a solution containing the respective probe (0.063 mM in Tris-HCl buffer, pH 8.00) directly onto poly(4-HPA)/GE surface at 42 °C for 20 min. This electrode is now called ssDNA/poly(4-HPA)/GE. Hybridization process was performed by adding the specific complement target in each case (polyCT-1 or polyCT-2, 20 μL 0.126 mM in Tris-HCl buffer pH 8.00) onto ssDNA/poly(4-HPA)/GE surface, keeping the electrode at 42 °C for 20 min. This electrode is now called dsDNA/poly(4-HPA)/GE. After each step, the electrodes were washed in 0.10 M phosphate buffer solution (PBS) under agitation for 6 s and dried under N_2(g)_ flow.

**Scheme 1:**
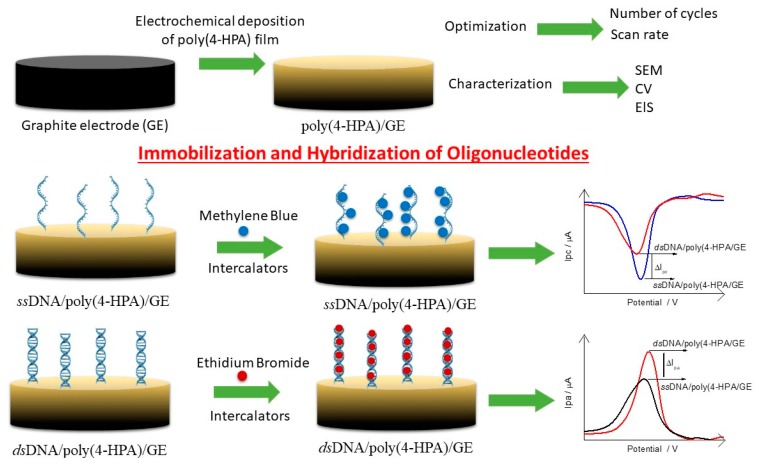
Proposed schematic for the biosensor development.

Methylene Blue (MB) or Ethidium Bromide (EB) intercalators were used to indirectly monitor the adsorption event of the ssDNA and dsDNA. In this case, 20 μL of the solution of one of the intercalators (0.50 mM containing 20 mM NaCl) were placed directly into contact with the biosensors surface the biosensors surface containing the probe (ssDNA/poly(4-HPA)/GE) and probe + target (dsDNA/poly(4-HPA)/GE) for 5 min, then washed again in PBS for 6 s, and dried with N_2(g)_. All the steps of the biosensor development can be summarized in Scheme 1.

### 2.7. Application of the Platform in the Immobilization and Detection of Mycobacterium tuberculosis

After the oligonucleotides detections, the proposed platform was applied in the detection of the preserved *Mycobacterium tuberculosis* genetic code, where the same standard procedure was used for the oligonucleotides immobilization was maintained (see Section 2.6). However, optimization studies for probe immobilization parameters and the target hybridization event were conducted to ensure a device with greater sensitivity and resolution. Table 1 displays all parameters investigated in the development of the genosensor. 

The development steps of the biosensor are similar to that shown in Scheme 1. However, the oligonucleotide was replaced by a specific *Mycobacterium tuberculosis* sequence and only methylene blue was used for monitoring the hybridization.

Using the optimal conditions for platform construction, the detection of a non-specific interferent of the *Mycobacterium tuberculosis* probe was carried out to verify the platform selectivity. 

## 3. Results and Discussion

### 3.1. Influence of the Scan Rate and Number of Cycles on 4-HPA Electropolymerization

**Figure 1:**
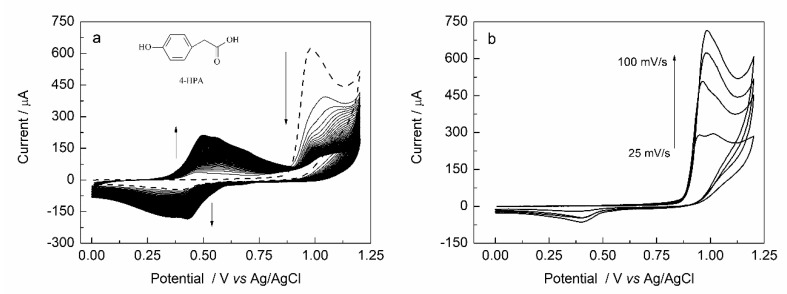
Cyclic voltammograms recorded in 0.5 M perchloric acid solution containing 2.5 mM 4-HPA: (**a**) 100 scans at 75 mV/s. (---) First scan. Arrows indicate the current direction; and (**b**) 1st scan at different scan rates (25, 50, 75, and 100 mV/s).

Figure 1a shows different CV scans for 4-HPA electropolymerization over GE surface. To all cases, it can be observed an irreversible oxidation peak at about +0.97 V, which is related to 4-HPA oxidation to a cation-radical, followed by oxygen evolution reaction at approximately +1.20 V. In the reverse scan there is a cathodic process at about +0.40 V, related with the reduction of adsorbed material onto electrode surface, probably after a chemical coupling. 

This analysis is consistent with a classic ECE mechanism: An electrochemical oxidation of the monomer to a cation radical followed by a chemical reaction resulting in a dimer and then, a second electrochemical process resulting from the reduction of the generated dimer. In the second scan, the corresponding oxidation process shows at about +0.53 V. The increase in the number of cycles results in an increase in the peak current values of the redox couple. Additionally, there is a decrease in the monomer oxidation peak current (*I*pa), together with a potential shift to a more anodic potential. This can be related to the consumption of the monomer close to the electrode surface for the formation and deposition of polymeric material derived from 4-HPA, which, in turn, makes the oxidation process more difficult to occur. As can be seen in Figure 1b the increase in the scan rate value is followed by a current increase in the monomer anodic oxidation because the diffusion layer growth closer to the electrode surface. The potential shift to more anodic potentials are not very significant and fast electron transfer kinetics is applied [37]. Experiments performed over GE with a different number of scans (25 to 200) presents the same behavior as the one in Figure 1a. 

### 3.2. Morphologic Studies

**Figure 2:**
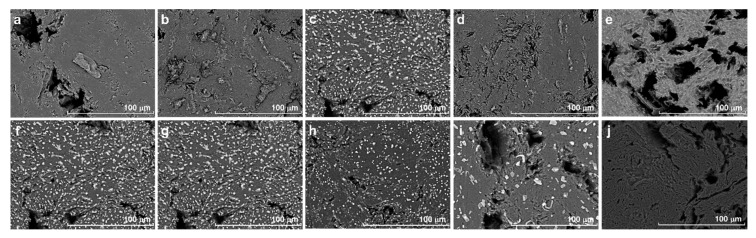
SEM images obtained for poly(4-HPA) films electropolymerized over graphite electrodes after (**a**) 25, (**b**) 50, (**c**) 100, (**d**) 150, and (**e**) 200 cycles of potential at 50 mV/s. The micrographs from f–i show the polymer films obtained with 100 cycles with different scan rates: (**f**) 25, (**g**) 50, (**h**) 75, and (**i**) 100 mV/s. The bare graphite electrode is also shown (**j**). Magnification of ×1000.

The morphology of bare GE and poly(2-HPA)/GE were analyzed by SEM (Figure 2). The micrographs from Figure 2a–e show the influence of the number of cycles in the electrogeneration of poly(4-HPA) and from Figure 2f–i the influence of scan rate.

The images obtained show that in all cases there is no total coating of the graphite surface, which has a high porosity. As the number of cycles of potential increases, more material is deposited. The electrode modified with 100 cycles seems more homogenously covered by a grain-like material. It is visible that with the increase of the scan rate there is a decrease of the electrode coverage visualized as the decrease of the amounts of grains over the electrode surface. At lower scan rates, more time is necessary to achieve the hundredth scan and the polymer can be formed more evenly.

### 3.3. Influence of Scan Rate and Number of Cycles on the Poly(4-HPA) Electrochemical Behavior 

**Figure 3:**
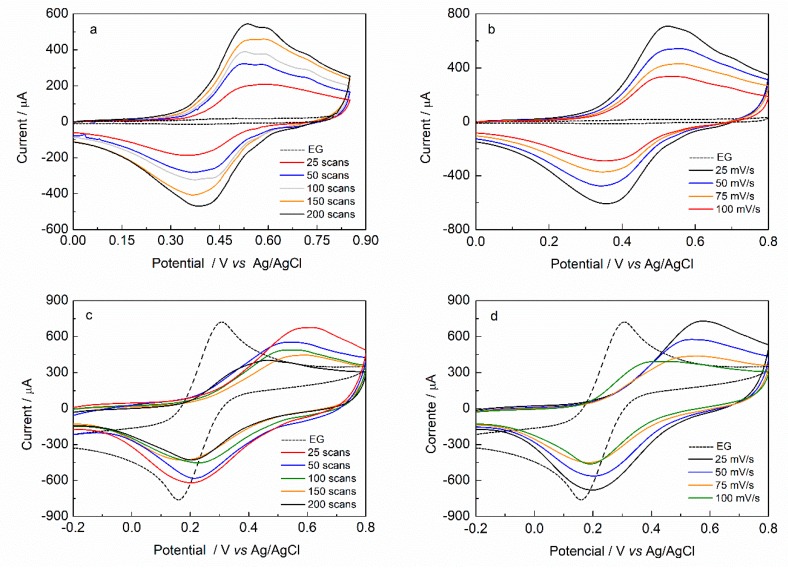
Cyclic voltammograms recorded in 0.5 M perchloric acid, 50 mV/s (**a**,**b**) and in 5.0 mM K_4_[Fe(CN)_6_]/K_3_[Fe(CN)_6_] containing 0.10 M KCl, 100 mV/s (**c**,**d**). Electrodes modified at (**a**–**c**) different number of scans (25, 50, 75, 100, 150, and 200) at a fixed scan rate of 50 mV/s and; and (**b**–**d**) different scan rates (25, 50, 75, and 100 mV/s) at a fixed number of scans of 100. (----) Cyclic voltammogram for GE.

The supporting electrolyte and a standard ferro/ferricyanide solution can provide useful electrochemical information besides serving to confirm GE modification in comparison with a non-modified GE. Figure 3 shows the electrochemical profile of the modified electrodes in the different number of cycles and scan rates, respectively, compared to the non-modified electrode in HClO_4_ solution (Figure 3a,b) and in ferro/ferricyanide solution (Figure 3c,d). 

From Figure 3a,b it is clear that GE surface is modified after all the electropolymerization experiments. An electroactivity at about +0.53/0.38 V can be observed. This behavior is at the same potential range as that presented during the electropolymerization process (Figure 1). This indicates that the material remains adsorbed onto GE surface even after washing steps. The polymeric film growth by the redox activity increase at +0.53/+0.38 V is observed as being influenced by the increase in the number of scans. Higher current values were observed to the modified electrodes with 200 cycles and smaller values to the electrodes with 25 cycles.

The current peak value for the electrode modified with 200 cycles is, approximately, 31% higher when compared to the electrode modified with 100 cycles. For the electrode modified with 25 cycles, the current peak value is, approximately, 36% smaller compared to the electrode modified with 100 cycles. Moreover, a higher peak current values were observed for the electrodes modified at 25 mV/s and smaller peak current values at 100 mV/s. The current peak increase for the electrode modified at 25 mV/s is 110% higher when compared to that one modified at 100 mV/s. These current peak values refer to the polymeric film electroactivity adsorbed onto GE surface.

The electroactivity of the polymeric films formed onto GE surface is associated with the aromatic rings superposition present on a 4-HPA structure [22]. In this way, a smaller scan rate tends to generate a more organized coupling, with a greater superposition of the rings, and consequently, a higher electroactivity of poly(4-HPA)/GE platform. On the other hand, higher scan rates tend to generate a coupling less organized, diminishing the film electroactivity. This analysis is in accordance with aniline electropolymerization [38].

Electroactive substances adsorbed onto an electrode surface result in alterations on the charge transfer properties of the system. The potassium ferrocyanide/ferricyanide complex has been largely used as a redox probe to investigate these alterations on the modified electrode charge transfer process. Possible electrostatic interactions between the redox probe and the polymer are capable to suggest information about poly(4-HPA) electric properties. According to Figure 3c,d, there is no full blockage on the charge transfer of the redox pair, but it can be observed an oxidation potential shift as well as a decrease in the faradaic current. This current decrease can indicate the adsorption of an anionic material. In this way, there is an electrostatic repulsion between the anionic redox probe and the polymeric film, decreasing the number of redox species reaching the electrode surface.

Figure 3c,d shows a similar behavior, with a redox current decrease and a shift to larger ΔE with all modifications performed over GE. As the amount of adsorbed material increases, a decrease in redox pair response is expected. Reduction values in the oxidation current of the redox pair by about 7%, 34%, and 46% were obtained for the electrodes modified with 25, 100, and 200 potential cycles, respectively, relative to the GE. The modifications in the electrochemical profiles with different scan rates confirm that the scan rate is a parameter that directly influences the charge transfer properties of the film. This result corroborates the discussion developed in Section 3.1. Higher scan rate tends to generate less efficient polymer coupling. This structural disorganization of the polymer allows the preserved functional groups to be present in the outermost layers of poly(4-HPA). According to the electropolymerization mechanism for poly(4-HPA) proposed by Rodrigues et al. [39], the higher electrostatic repulsion of the anionic pair can be explained by the presence of negative charges of the carboxyl group preserved in the outermost regions of the film. 

### 3.4. Electrochemical Impedance Spectroscopy Studies for the Transducer

**Figure 4:**
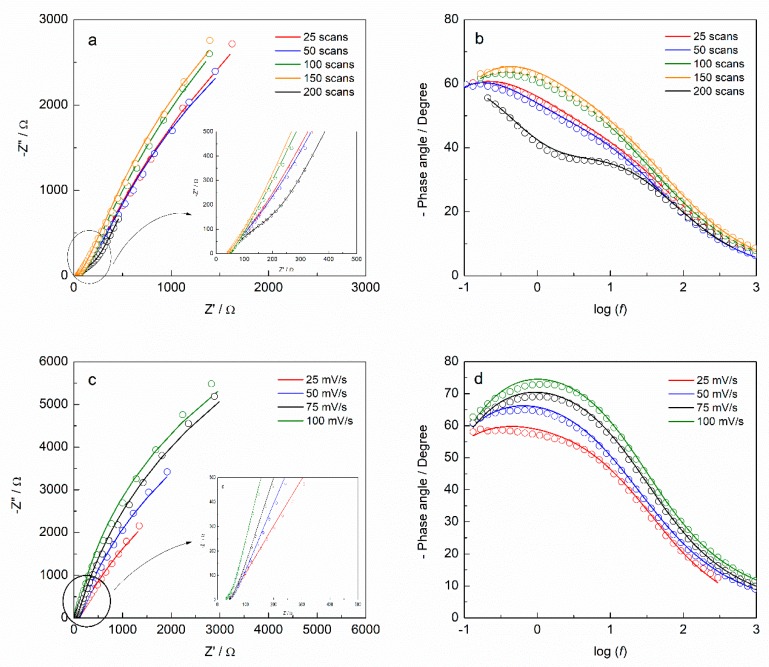
Nyquist (**a**–**c**) and Bode (**b**–**d**) diagrams for poly(4-HPA)/GE obtained in 5.0 mM K_4_[Fe(CN)_6_]/K_3_[Fe(CN)_6_] containing 0.10 M KCl. (**a**,**b**) the electrodes modified with 25, 50, 100, 150, and 200 scans. (**c**,**d**) poly(4-HPA)/GE platforms modified at 25, 50, 75, and 100 mV/s. Amplitude 10 mV; frequency range: 10^6^ to 10^−2^ Hz. Solid lines represent the circuit’s fit to the experimental data.

Figure 4 shows the electrochemical impedance spectra obtained at OCP (+0.23 V) of the poly(4-HPA)/GE platforms obtained under the different studied conditions.

EIS is one of the most powerful and sensitive techniques for investigating the electrical properties of the surface of modified electrodes since it allows obtaining information from different interfacial electrochemical processes and their time constants. For all cases, the experimental plot of the complex plane presents a straight line of approximately 45° in the high frequencies domain, characteristic behavior of the diffusional process on rough surfaces. This straight line is followed by a poorly defined semicircle in the low frequency domain, which represents a behavior of high resistance to charge transfer. This spectrum profile characterizes a system controlled both by mass transport in the high frequencies and by electronic transport in the low frequencies.

An equivalent circuit described as R_s_(Q_dl_R_ct_)(Q_f_[R_f_W]) was used for experimental data simulation based on equivalent circuits for conductive polymers presented in the literature. The proposed circuit is an adaptation to the Randle’s circuit. The equivalent electrical circuit consists of an ohmic element representing the solution resistance (R_S_), followed by two parallel arrangements of capacitive (Q), resistive (R), and diffusive (W) elements. While the elements Q_dl_ and R_ct_ represent the interfacial pseudo-capacitance and the charge transfer resistance at the equilibrium potential, respectively. The element Q_f_ represents the pseudo-capacitance and the element R_f_ represents the resistance to ionic transport, both associated with the charge/discharge of the adsorbed film. W element refers to the semi-infinite linear diffusion phenomenon occurring at the electrode/solution interface. The necessity of the use of two components for the polymerized films suggests the formation of a highly rough/porous polymeric film of variable length formed by long and short chains alternated.

Different parameter values for the equivalent circuit obtained from numeric simulation are summarized in Table 2 and Table 3. In agreement with the CVs in potassium ferrocyanide/ferricyanide solution (Figure 3), it can be noted that as the number of cycles or the scan rate increases, the poly(4-HPA)/GE platform tends to show a higher total resistance (R_T_ = R_ct_ + R_f_) for the redox pair studied. 

The platform modified with 200 cycles presented a total resistance 21% higher when compared to the platform modified with 100 cycles. On the other hand, the modified platform with 25 cycles presented a total resistance of 13% lower than the modified platform with 100 cycles. The platform modified at 100 mV/s showed a total resistance 49% higher when compared to the platform modified at 50 mV/s. On the other hand, the platform modified at 25 mV/s presented a total resistance 22% lower than the platform modified at 50 mV/s.

The R_ct_ obtained in the OCP acts as an indirect measure of the exchange current density (Io = R_T_/nFR_ct_) for the outer sphere redox reaction at the modified electrode/solution interface. The results show that different electropolymerization parameters exert a strong influence on the charge transfer properties of the electrode surface. This is related to the different thicknesses that are obtained by varying both the number of cycles and the scan rate. While the thickness of thin film has a non-uniform distribution [40], it is well established that the film resistance (R_f_) is directly proportional to its thickness, while the pseudo-capacitance is inversely proportional [35,41]. 

In this way, the results obtained showed that a larger number of cycles lead to thicker films, which is justified by the greater amount of deposited material. In addition, higher scan rate was responsible for thicker films formation, which is attributed to the disorganization during the aromatic rings coupling. These data corroborate with those found in CV studies and the circuits presented reasonable chi-square (χ^2^) values, in the order of 10^−3^. The system is then stable over time and presents a good experimental data adjustment, supporting a low statistical error on the experimental data simulation.

### 3.5. Influence of the Electropolymerization Parameters on the Adsorption and Detection of Oligonucleotides

The electrodes modified with the different number of cycles and different scan rates were evaluated with the immobilization of polyGA-1 and polyGA-2 probes, respectively, as well with the intercalation with the complementary polyCT-1 and polyCT-2 targets, respectively. 

**Figure 5:**
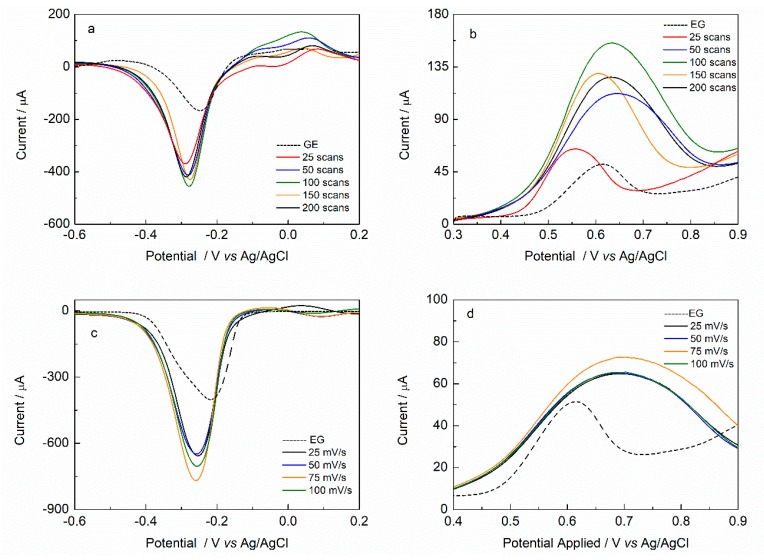
Differential pulse voltamograms recorded in a 0.10 M phosphate buffer solution, pH 7.60 of poly(4-HPA)/GE. (**a**–**c**) Methylene blue reduction containing probe and (**b**–**d**) Ethidium bromide oxidation containing probe + target. Amplitude: 25 mV; pulse period: 0.20 s; pulse height: 0.06 s; and ν = 20 mV/s. (---) DPV for GE.

Figure 5a shows the indirect monitoring of the adsorption of ssDNA using the AM reduction, and Figure 5b shows the hybridization monitoring using the indirect detection of the BE oxidation for the transducers obtained by the electropolymerization of the 4-HPA with different potential cycles. Figure 5c,d show the same monitoring but for those poly(4-HPA) platforms obtained at different scanning rates.

The use of MB and EB is very common in electrochemical to monitor the hybridization event. Such compounds can bind to DNA by reversible physical intercalation between base pairs or by electrostatic interaction at specific sites of the nitrogenous bases [27]. MB is an organic dye belonging to the phenothiazine family, interacts with DNA through electrostatic interactions or by preferential binding to free guanine bases in ssDNA [42,43]. The orientation and conformation of the MB-guanine complexes were determined in three ways: T-shaped, nonstacked, and face-to-face [44]. The difference between electrostatic attraction to DNA and real intercalation is the ionic strength. The simple addition of NaCl above 10 mM [43] is enough for the intercalation to prevail. In this work, we used 20.0 mM. On the other hand, the intercalation of the EB to the DNA occurs through the stacking of the planar aromatic groups between the base pairs spaces. For an intercalator to fit between the base pairs, the bases have to separate, distorting the DNA strands, by unwinding the double strand.

As can be seen in Figure 5a,c (both using MB) the higher current peak for dsDNA (and higher Δ*I* compared to ssDNA) was obtained with the polymer formed with 100 cycles at a scan rate of 75 mV/s. The same result was obtained from Figure 5b,d (both using EB). Since the *I*pa and *I*pc are directly proportional to the analyte concentration, the responses point to an accumulation of these intercalators over the modified electrodes.

Table 4 and Table 5 shows the peak current values obtained for the modification studied. It is possible to observe that the GE presented the worst results. This observation shows that the presence of the poly(4-HPA) tends to improve the biological material adsorption onto the electrode surface, and consequently it is a good transducer for the development of genosensors.

### 3.6. Investigation of Poly(4-HPA)/GE Platform Use in the Immobilization, Optmization, and Detection of Oligonucleotide Specific for Mycobacterium tuberculosis 

The poly(4-HPA)/GE platform formed at 75 mV/s with 100 scans using MB (higher current responses) presented the best results for the oligonucleotide immobilization and it was used for the hybridization experiment with a complementary (preserved genetic code of the *Mycobacterium tuberculosis* bacillus) target. 

Optimizations were carried seeking the biosensor better performance as shown in Table 1. It was found that 0.15 M PBS at pH 7.6 is the better solution for the experiments. The buffer concentration is directly related with the mimicry of the biologic environment and pH plays a pivotal role in DNA maintenance, once that the pH value near physiologic ensures that the biological material will not be denatured. For immobilization, a 15 µL of the probe solution over the electrode surface for 20 min at 42 °C is also the best setup for the proposed biosensor. For hybridization, a 10 µL of target solution over the biosensor surface for 20 min at 42 °C was the optimum conditions.

**Figure 6:**
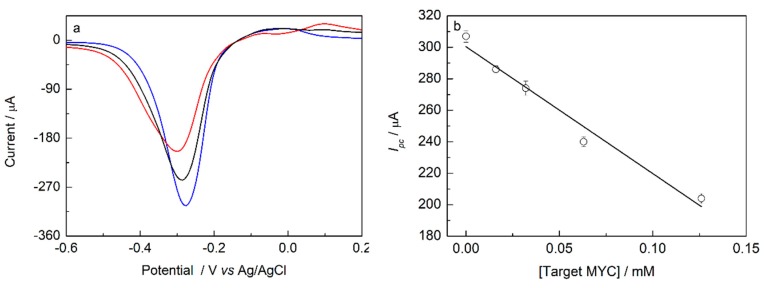
(**a**) Differential pulse voltamograms for methylene blue reduction content: (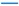) probe; (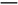) probe + interferent; and (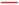) probe + specific target. Amplitude: 25 mV; pulse period: 0.20 s; pulse height: 0.06 s; and ν = 20 mV/s. (**b**) Analytical curve obtained for the biosensor (mean ± SD, n = 3), being evaluated the target concentrations of: 0.000, 0.016, 0.032, 0.063, and 0.126 mM using a solution volume of 10 μL, hybridization time of 20 min and incubation temperature of 42 °C. Intercalator: 20 μL of AM 5.00 × 10^−4^ M.

Figure 6a shows the VPD for the biosensor response to *Mycobacterium tuberculosis* probe (MYC), probe + specific target and probe + interferent, as well as the variation of *I*pc as a function of the concentration of the target solution, under the best conditions of the response of the genossensor. It is observed that the hybridization resulted in a current decrease because of the unavailability of free guanine bases to interact with MB, as was predicted. In addition, the sensitivity of the device in the presence of non-specific targets is an important factor to guarantee the system specificity. In this way, the sensitivity of the biosensor to its non-specific target response was evaluated, with a sequence that presents seven mismatches in it’s sequencing, which represents 65% of similarity to the specific target.

It is observed from Figure 6a that the interferent caused a 16% (±1.4 n = 3) decrease in the probe signal. Since this interferent has 65% (±5.2 n = 3) complementarity with the probe ssDNA, this result is satisfactory since the signal decrease was 46% (±2.9 n = 3) lower than the decrease caused by the specific target. This decrease in probe signal is justified by the regions of the interferent that have complementarity with the probe, which may lead to partial hybridizations in certain parts of the genetic code.

The sensitivity of the biosensor was investigated (Figure 6b) using the optimum conditions for immobilization and hybridization. In this study, the concentration of the target solution was changed. As shown in Figure 6b, a linear relationship in this concentration range studied was observed. The equation obtained from the linear regression was: *I*pc (μA) = 300.41 − 806.11 [target] (mM) with linear correlation coefficient r^2^ = 0.987. The limit of detection (LOD = 3 σ/S) was 0.56 (±0.05) μM and the limit of quantification (LOQ = 10 σ/S) was 8.6 (±0.7) μM. As the objective of the device is to recognize the target, i.e., to provide information about the infection or not by the bacillus, we understand that the results found here are considered promising since the developed genosensor presented reproducibility, stability, and was able to detect a very low quantity of the target.

## 4. Conclusions

This work confirmed the efficiency of the poly(4-HPA) platform for the development of biosensors. It was possible to observe significant improvements for the modified electrodes, both in the immobilization and in the detection of the oligonucleotides, when compared to the unmodified electrodes. From the electropolymerization studies of the 4-HPA on GE, as well as the oligonucleotides incorporation on poly(4-HPA) matrix, it can be concluded that the platform poly(4-HPA)/GE with 100 cycles at 75 mV/s presented the best characteristics for application in the development of genosensors. The system was applied in a real sequence and it was possible to differentiate the genetic codes preserved from the *Mycobacterium tuberculosis* bacillus, its specific complementary target and interfering with 65% of complementarity.

## Figures and Tables

**Table 1:** Parameters investigated for construction of the genosensor.

Parameter	Variations
Buffer solution	Acetate, Phosphate, Tris-HCl
Concentration/M	0.05; 0.10; 0.15
pH	7.0; 7.2; 7.4; 7.6; 7.8; 8.0
Probe volume/μL	5; 10; 15; 20
Target volume/μL	5; 10; 15; 20
Immobilization temperature/°C	32; 42; 52
Hybridization temperature/°C	32; 42; 52
Immobilization time/min	10; 15; 20; 25
Hybridization time/min	10; 15; 20; 25

**Table 2:** Parameters obtained from electrochemical impedance spectroscopy data simulation for poly-(4-HPA)/GE platforms modified with different numbers of cycles using the equivalent circuit proposed (mean, n = 3).

Parameter	Number of Cycles				
	25	50	100	150	200
R_s_ (Ω)	43.9	46.6	40.4	45.9	44.9
R_ct_ (Ω)	211	267	305	403	498
Q_dl_ (mΩ^−1^ s^n^)	1.13	1.05	1.13	1.38	0.683
n_dl_	0.58	0.60	0.56	0.52	0.65
R_f_ (kΩ)	10.3	10.7	11.8	12.9	14.1
Q_f_ (µΩ^−1^ s^n^)	623	520	339	316	216
n_f_	0.83	0.85	0.89	0.87	0.81
W (mΩ^−1^ s^0.5^)	0.52	0.94	1.24	0.82	0.93
χ^2^ × 10^−3^	9.5	8.8	7.6	8.0	9.8

**Table 3:** Parameters obtained from electrochemical impedance spectroscopy data simulation for poly-(4-HPA)/GE platforms modified with different scan rates (mean, n = 3).

Parameter	Scan Rate (mV/s)			
	25	50	75	100
R_s_ (Ω)	44.1	44.4	43.1	43.9
R_ct_ (Ω)	309	319	427	489
Q_dl_ (mΩ^−1^ s^n^)	1.42	1.34	6.24	6.24
n_dl_	0.61	0.52	0.30	0.29
R_f_ (kΩ)	9.48	12.2	16.7	18.3
Q_f_ (µΩ^−1^ s^n^)	488	293	176	181
n_f_	0.83	0.89	0.92	0.87
W (mΩ^−1^ s^0.5^)	0.94	1.27	0.83	1.14
χ^2^ × 10^−3^	14.4	9.1	13.0	9.7

**Table 4:** Current peak values obtained from MB reduction and EB oxidation for GE and poly(4-HPA)/GE polymerized at different potential cycles containing the immobilized oligonucleotide (mean, n = 3).

Transducer	*I*pc for MB/µA	*I*pa for EB/µA
GE	166	51
poly(4-HPA)/GE; 25 cycles	369	64
poly(4-HPA)/GE; 50 cycles	412	112
poly(4-HPA)/GE; 100 cycles	455	155
poly(4-HPA)/GE; 150 cycles	430	129
poly(4-HPA)/GE; 200 cycles	419	126

**Table 5:** Current peak values for MB reduction and EB oxidation for GE and poly(4-HPA)/GE modified at different scan rates containing the immobilized oligonucleotide (mean, n = 3).

Transducer	*I*pc for MB/µA	*I*pa for EB/µA
GE	401	51
poly(4-HPA)/GE; 25 mV/s	648	65
poly(4-HPA)/GE; 50 mV/s	657	65
poly(4-HPA)/GE; 75 mV/s	768	73
poly(4-HPA)/GE; 100 mV/s	704	65
